# Favoring recruitment as a conservation strategy to improve the resilience of long‐lived reptile populations: Insights from a population viability analysis

**DOI:** 10.1002/ece3.8021

**Published:** 2021-09-15

**Authors:** Chloé Warret Rodrigues, Baptiste Angin, Aurélien Besnard

**Affiliations:** ^1^ Office National de la Chasse et de la Faune Sauvage Cellule Technique des Antilles françaises Trois‐îlets France; ^2^ Department of Biological Sciences University of Manitoba Winnipeg Canada; ^3^ Ardops Environnement Les Abymes France; ^4^ CEFE Univ Montpellier CNRS EPHE‐PSL University IRD Univ Paul Valéry Montpellier 3 Montpellier France

**Keywords:** canalization hypothesis, Capture–recapture, Lesser Antillean iguana, population dynamics, recruitment, wildlife management

## Abstract

In long‐lived species, although adult survival typically has the highest elasticity, temporal variations in less canalized demographic parameters are the main drivers of population dynamics. Targeting recruitment rates may thus be the most effective strategy to manage these species. We analyzed 1,136 capture–recapture histories collected over 9 years in an isolated population of the critically endangered Lesser Antillean iguana, using a robust design Pradel model to estimate adult survival and recruitment rates. From an adult population size estimated at 928 in 2013, we found a yearly decline of 4% over the 8‐year period. As expected under the canalization hypothesis for a long‐lived species, adult survival was high and constant, with little possibility for improvement, whereas the recruitment rate varied over time and likely drove the observed population decline. We then used a prospective perturbation analysis to explore whether managing the species’ immature cohorts would at least slow the population decline. The prospective perturbation analysis suggested that a significant and sustained conservation effort would be needed to achieve a recruitment rate high enough to slow the population decline. We posit that the high recruitment rate achieved in 2014—likely due to the maintenance in 2012 of the main nesting sites used by this population—would be sufficient to slow this population's decline if it was sustained each year. Based on the results of diverse pilot studies we conducted, we identified the most likely threats targeting the eggs and immature cohorts, stressing the need to improve reproductive success and survival of immature iguanas. The threats we identified are also involved in the decline of several reptile species, and species from other taxa such as ground‐nesting birds. These findings on a little‐studied taxon provide further evidence that focusing on the immature life stages of long‐lived species can be key to their conservation.

## Graphical abstract

In long‐lived species, under normal conditions, adult survival is fairly high and constant, whereas less canalized demographic traits (e.g. the recruitment rate) vary over time and drive population trajectories. We used a long‐term set of capture–recapture data collected on a population of the critically endangered Lesser Antillean iguana, and show that the current demographic parameters will lead that population to quasi‐extinction. We also predicted population trajectories under different scenarios to forecast the magnitude of conservation effort that could halt the population decline. We finally review the threats to preadult stages and proposed actions to improve recruitment rates. Our study provides further evidence, in an understudied taxon, that managing immature stages can be key to the conservation of long‐lived species.

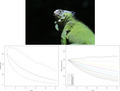

## 1 INTRODUCTION

The population growth rate is central to population ecology and conservation (Sibly & Hone, 2002; Sim et al., 2011). It reflects the variation in population abundance under specific conditions and is an endpoint measurement of population viability (Reed, 2005). A principal goal of conservation strategy is therefore to increase, or at least maintain, the growth rate of endangered wildlife populations (Sibly & Hone, 2002). Achieving this goal requires a detailed understanding of the demographic processes causing population decline and how the population growth rate is affected by variations in vital rates and demographic parameters (e.g., survival by age class or recruitment) (Caswell, 2000). Typically, there are two complementary ways to gain such knowledge. Retrospective analyses measure how variations in demographic parameters affected the growth rate of a given population, whereas prospective perturbation analyses model the relative contribution of demographic parameters to future asymptotic population growth rates (Caswell, 2000). Acting on the demographic parameters to which the population growth rate is the most sensitive is often considered the most efficient management strategy (Manlik, 2019; Mills et al., 1999; Reed et al., 2002). However, basing conservation actions on modeled perturbations may lead to unrealistic goals (Caswell, 2000; Manlik, 2019). When conducting population viability analyses, conservationists must take into account the specific characteristics of the target population and set realistic perturbations in their models to guide population management.

In long‐lived species, the adult survival rate is usually the demographic parameter with the highest elasticity (i.e., contributing the most to the population growth rate at equal proportional perturbation) and thereby contributes greatly to population viability (Gaillard et al., 1998; Sæther et al., 2013). However, because of its strong influence on the asymptotic population growth rate, adult survival is also expected to be the demographic parameter with the lowest variability. The canalization hypothesis states that under natural selection processes, the variability of a trait is negatively correlated to the trait's impact on average individual fitness (Gaillard & Yoccoz, 2003; Péron et al., 2016). Thus, in long‐lived species, traits favoring adult survival are canalized against environmental (and genetic) perturbations, leading to both low temporal variability and interindividual variance in this parameter (Gaillard & Yoccoz, 2003; Gibson & Wagner, 2000; Péron et al., 2016). As a result, adult survival may not contribute significantly to population dynamics in long‐lived species compared with reproduction and the demographic parameters of immature individuals. The wide natural fluctuations of the less canalized demographic parameters and the high number of juveniles result in their larger contribution to fluctuations in population abundance and thus population trajectories (Gaillard & Yoccoz, 2003; Manlik, 2019).

A strong implication of the canalization hypothesis is that improving the population conservation status in long‐lived species may not be responsive to management actions targeting adult survival if this parameter is already high and fairly constant. Conversely, focusing conservation actions on improving the less canalized demographic parameters may prove more effective at halting or even reversing population decline (Manlik, 2019; Mortensen & Reed, 2016; Sergio et al., 2020). A growing body of literature emphasizes the key role of reproduction and juvenile demographic parameters in long‐lived species conservation. Empirical studies on many species have supported the canalization hypothesis including in birds and both terrestrial and marine mammals, confirming the key role of the more variable demographic parameters in driving population dynamics (Beston, 2011; Couet et al., 2019; Fay et al., 2015; Gaillard et al., 1998; Genovart et al., 2018; Manlik et al., 2016; Mortensen & Reed, 2016). As is often the case in conservation research, these studies are taxonomically biased toward birds and mammals, while reptiles are underrepresented (Di Marco et al., 2017). Yet, reptiles, and specifically herbivorous reptiles, have among the highest proportion of threatened species in comparison with other vertebrates (Atwood et al., 2020). Understanding the contribution of different traits to population dynamics is crucial to address conservation issues in these highly threatened species.

The Lesser Antillean iguana (*Iguana delicatissima*) is a long‐lived reptile endemic to the Lesser Antilles. It is critically endangered, having experienced a substantial range contraction and a population decline of at least 70% since Europeans settled in the area during the 17th and 18th centuries (Knapp et al., 2014; van den Burg et al., 2018). In the French West Indies, the current main threat to the species is competition and hybridization with the green iguana (*Iguana iguana*) (Breuil, 2013; Knapp et al., 2014; Vuillaume et al., 2015). Most Lesser Antillean iguana populations have been extirpated due to genetic admixture with the invasive and larger green iguanas, which outcompete *I. delicatissima* males for reproduction (Vuillaume et al., 2015). Introduced predators and habitat degradation—a concern for island fauna worldwide—are also a major threat for some populations of Lesser Antillean iguanas across its range (Knapp et al., 2014). Rodents, including rats (*Rattus* spp.), and small carnivores such as the small Indian mongoose (*Herpestes auropunctatus*) are among the introduced predators with the most impact, responsible for a majority of extinctions of island species worldwide (Case & Bolger, 1991). In terms of habitat degradation, pastoralism is a major anthropogenic disturbance, threatening wildlife through diverse mechanisms (Hailey et al., 2011; Madhusudan, 2004). Livestock may trample nests when foraging, a concern both for reptiles and for ground‐nesting birds (Campbell et al., 2020; Fan et al., 2020; Mandema et al., 2013). Additionally, high grazing pressure prevents shrub regeneration, the establishment of seedlings, and their subsequent recruitment into the mature population (Tiver & Andrew, 1997). The resulting lack of vegetation cover increases the susceptibility to predation of animals that depend on the understory to escape predators, as well as create competition with sympatric herbivores feeding on shrubs and seedlings (Mishra et al., 2002; Smelansky & Tishkov, 2012). High grazing pressure may also cause soil erosion (Evans, 1997), which may directly impact females by decreasing the availability of suitable ground for nesting.

To address the threats to the Lesser Antillean iguana, a large conservation program was initiated in 2010, with the launch of a French National Action Plan (Legouez, 2010). The plan included a long‐term standardized survey of the relictual populations inhabiting satellite and smaller islands of Guadeloupe and Martinique, including Chancel Islet. Chancel is a privately owned islet off Martinique where a population of the native iguana remains isolated from the green iguana. The islet's small size, its relatively sparse vegetation cover, and the absence of major threats to the adult population provide an ideal situation to ensure the conservation of the species and study its demography.

Here, we modeled data from the capture–recapture monitoring program conducted between 2012 and 2020 on Chancel Islet to estimate adult survival and recruitment. The latter is a combination of both reproduction parameters and juvenile survival (Gaillard et al., 1998; Reed et al., 2003). Based on the canalization hypothesis and the lack of apparent threat to adult survival on the islet, we predicted that: (1) adult survival is high and constant between years and (2) recruitment is the main driver of population dynamics and therefore offers more opportunities for conservation actions. We tested these predictions using a retrospective analysis and a prospective perturbation analysis through a population viability analysis (PVA). The PVA allowed us to explore the extent to which adult survival and recruitment must be improved to ensure the long‐term persistence of this population. Additionally, using selected literature, the results of pilot studies, and empirical observations, we identified the main potential threats and actions that could be implemented to improve this population's conservation status, which should be applicable to long‐lived squamates in general.

## 2 MATERIALS AND METHODS

### 2.1 Study site

**FIGURE 1:**
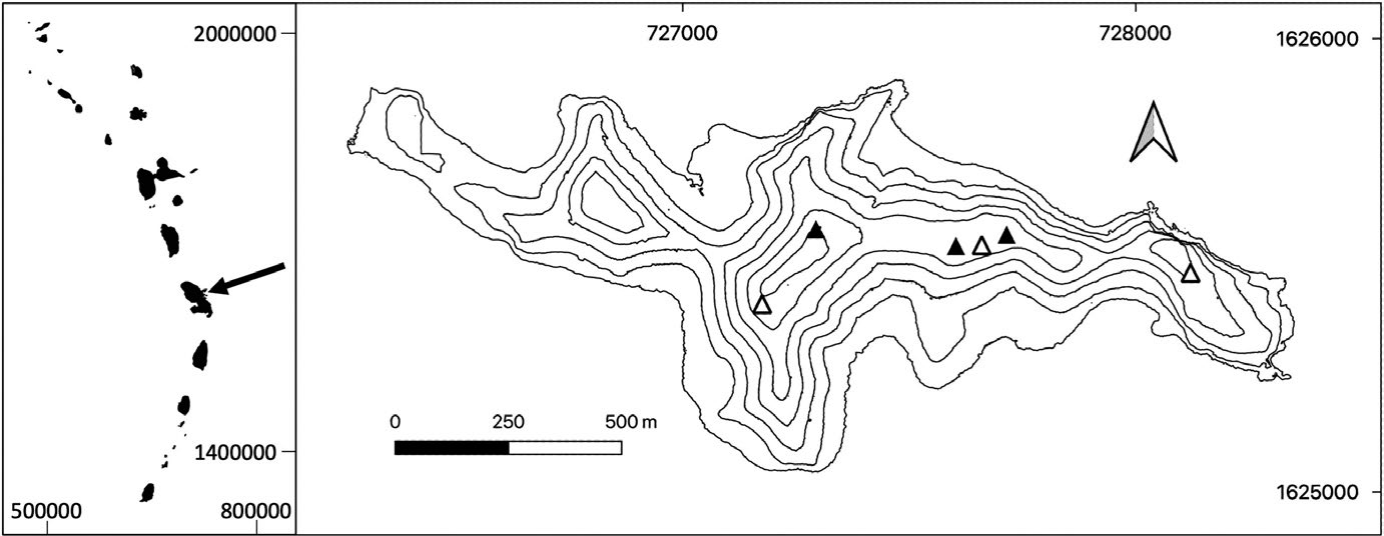
Location of Chancel Islet in the Lesser Antilles (black arrow, left panel), and map of Chancel Islet with major (black triangles) and minor (white triangles) known nesting sites

The study was conducted off the east coast of Martinique (Lesser Antilles) on Chancel, an offshore islet in Le Robert Bay (Figure 1). This small islet (0.7 km^2^) has a maximum altitude of 68 m and is separated from the coast by a 300‐m sea channel. Its vegetation is characterized by xeric shrubland and coastal forests with a few patches of mangrove. Fifty plant species have been recorded, dominated by four tree species: *Tabebuia heterophylla*, *Coccoloba uvifera*, *Haematoxylum campechianum*, and *Hippomane mancinella* (Delnatte, 2020). Chancel is a private islet, but has been protected by a biotope preservation decree issued by the local jurisdiction of Martinique since 2005 to favor the conservation of the Lesser Antillean iguana's habitat. The only year‐round inhabitant of Chancel is its owner, who is actively favorable to research and conservation. However, despite the protection decree, some threats to Chancel's habitats remain. Two small parts of the islet remain open for tourism, but the combined size of both areas is limited (<5% of the total surface area of the islet), so the negative effects of tourism are likely negligible at the scale of the iguana population. The larger threat is likely to be livestock: a flock varying yearly from 50 to 500 sheep ranges freely on the islet. High grazing pressure negatively impacts the understory, prevents forest regeneration, and favors soil erosion (Figure S1).

### 2.2 Data collection

Between 2012 and 2020, we conducted a yearly mark–recapture survey at the end of the males’ reproductive peak of activity (i.e., 9 primary sessions). Each primary session consisted of five secondary sessions (i.e., 5 consecutive days of sampling for 7.5 hr per day). We thus sampled the population over 45 secondary sessions. The capture teams consisted of 10 to 12 people, at least half of whom were proficient at spotting and capturing iguanas. Field assistants were divided into two‐person teams, with each inexperienced person partnered with someone experienced. To ensure comprehensive and uniform coverage of the islet, we divided it into five zones that could be covered in a similar duration. Consequently, zones with dense cover or high iguana density were smaller than open zones or those with few iguanas. We minimized observer bias by randomly assigning a two‐person team to a zone at the start of the five‐day sample period. Each two‐person team systematically searched for iguanas in their zone before moving to the next zone on the following day. Therefore, all teams had the opportunity to search each zone for a total of 7.5 hr. We captured iguanas by hand or with a noose and scanned them for an existing PIT tag (11.5 × 2.12 mm; 0.1 g, Trovan^®^ Ltd., EID, USA); if they lacked a tag, we inserted a new one on the ventral side of the right thigh.

Upon capture, we recorded the sex based on the conspicuous sexual dimorphism of the species (e.g., enlarged nuchal–dorsal crest scales, or the bilateral bulge from the hemipenes at the base of the tail in males) (Knapp et al., 2014), total and snout–vent length (SVL), and body mass. Experienced members of the team also assessed whether females were gravid by gently palpating the abdomen. Each individual was assigned as an adult (SVL > 22 cm), subadult (18 cm < SVL < 22 cm), or juvenile (SVL < 18 cm) based on morphological criteria (e.g., juvenile body color is bright green, with white stripes on the proximal end of the lower jaw: Individuals darken as they age) and SVL (Knapp et al., 2014). For this study, adult and subadult iguanas were considered “adults.” To prevent immediate physical recapture, before release we identified each iguana with a unique alphanumeric code on both sides of its body, using a water‐based marker. These marks have no impact on the iguanas' health and generally fade quickly after a survey (Warret Rodrigues and Angin, pers. obs.). The capture program and handling methods were approved by Martinique's Agency for the Environment, Development and Housing, that is, the governmental authority in charge of implementing the National Action Plan. The primary sessions were carried out under the permits #041‐0001, #2013085‐009, #201604‐0001, #201703‐0006, #02‐2018‐02‐16‐002, and #02‐2019‐03‐13‐003.

### 2.3 Statistical analysis

We analyzed the capture–recapture data using a robust design Pradel model, which is based on two nested temporal scales of sampling: the year and the repeated intra‐year sessions (Pradel, 1996). Robust design schemes allow the population to be open between the primary sessions, but assume closure for the secondary sessions (Kendall et al., 1997). Chancel Islet is geographically closed, with migration events in and out of the islet unlikely. As this species is long‐lived, it was reasonable to assume population closure over the five consecutive days of our secondary sessions. The Pradel models allow the simultaneous estimation of survival probability, recruitment rates, capture and recapture probabilities, and population size. Recruitment is defined as the number of adults that recruit into the population in a given year for any adult present in the population the year before (Pradel, 1996). We analyzed only adult data because juvenile Lesser Antillean iguanas (SVL < 18 cm) usually have substantially lower capture and recapture probabilities than adults. They represented only 0 to 0.7% of total captures per year and thus were too few to model age‐specific parameters. Their inclusion could have generated strong recapture heterogeneity, leading to biased estimates of demographic parameters (Arsovski et al., 2018).

As goodness‐of‐fit (GOF) tests are not available for robust design data types, we performed a GOF test for the Cormack–Jolly–Seber models on a dataset in which data of the same year were merged. This GOF test was performed using U‐care (Choquet et al., 2009), and its high significance suggested capture heterogeneity (see “Results”). To deal with this source of overdispersion, we compared models with and without capture heterogeneity using the Akaike information criteria with correction for small sample size (AICc) (Hurvich & Tsai, 1989). Models with capture heterogeneity had substantially lower AICc than models without heterogeneity (see “Results”). Yet, we do not know whether—or to what extent—potentially remaining overdispersion biased the precision of our estimates.

We first fitted a full model with yearly variations and a sex‐dependent effect on survival and recruitment. Capture and recapture probabilities were modeled allowing for yearly variation, sex, and heterogeneity effects. We then simplified the model based on the AICc. If two models were less than two AICc points apart, we selected the most parsimonious one. We simplified the capture and recapture probabilities by first removing the sex effect, then heterogeneity, and finally yearly variation. Using our best model after modeling capture and recapture, we then simplified recruitment by removing sex and yearly variation, in that order, and then simplified survival probability with the same procedure.

All analyses were conducted using the MARK program (White & Burnham, 1999). We provide the estimates with their 95% confidence interval (CI).

### 2.4 Population trajectory simulation

To predict the population trajectory over the next 50 years, we simulated 1,000 trajectories using the survival probability and recruitment estimated in the previous step. To take into account recruitment variation over the years, we randomly sampled one of the estimated recruitment values at each time step after excluding the first two years, which may be biased by the low number of captured and marked individuals (see “Results”). We compared the population trajectory predicted with estimated recruitment with trajectories simulated by increasing recruitment up to 40% by 5% increments. We also compared the population trajectory predicted when increasing adult survival from 0.85 (estimated adult survival; see “Results”) to 0.90 by 1% increments. The initial population size was fixed to 610 (i.e. the population size estimated in 2020 – see “Results”). We simulated demographic stochasticity by random trial in a binomial distribution for survival and Poisson distribution for recruitment. Simulations were conducted using R (R Core Team, 2019).

### 2.5 Nesting site monitoring

On the islet, the state of Lesser Antillean iguana nesting sites has been raised as a conservation concern. Due to many years of erosion, the soil of the nesting sites has become rocky and hard to dig, likely favoring predation, competition for space between females, and nest collapse (unpublished data). In the context of a pilot study to assess the effect of nesting site maintenance on reproductive success, we monitored the three main nesting sites before (in 2012) and after (in 2013) fixing the fences, turning the soil, and removing stones to obtain a layer of about 50 cm of loose soil. On each site, before and after this maintenance, we counted the number of nests, individually marked them, and counted the number of eggs scattered on the surface. Scattered eggs were removed from the nesting sites to avoid counting them more than once. After hatching occurred, we assessed the success of each nest by excavating the clutches to determine the fate of each egg. We only recorded clutch data for the nests that were successfully excavated, that is, for which we are confident that we retrieved the entire clutch (*N*_2012_ = 3 and *N*_2013_ = 5). We recorded egg data from scattered eggs and excavated nests.

## 3 RESULTS

Over the nine monitoring years, there were a total of 3,339 capture events of 1,156 individuals. An individual was on average captured 2.89 (±*SD* = 2.52) times. These 1,156 individuals included 5 hatchlings, 602 females, 533 males, and 16 iguanas of undetermined sex. We only analyzed the capture–recapture histories of the 1,135 sexed individuals. At least 25% of our recaptures between 2019 and 2020 were 10 years old or over, suggesting an aging population. In 2017 and 2018, we recaptured 2 adults (SVL of 23 cm and 27 cm) first marked in 1997 (Breuil and Day, unpublished results), which we estimate to be at least 23 and 24 years old, confirming that the presumed longevity in this species under natural conditions exceeds 20 years (Knapp et al., 2014).

### 3.1 Retrospective analysis

The GOF test was highly significant (χ^2^ = 166.60, *df* = 46, *p* < .001), and both transience and trap dependence were also highly significant (χ^2^ = 73.30, *df* = 7, *p* < .001 and χ^2^ = 30.31, *df* = 6, *p* < .001, respectively), suggesting strong capture heterogeneity (Pradel et al., 2005). As suspected, models with capture heterogeneity had substantially lower AICc (see Table 1).

**TABLE 1:** Model selection statistics for the capture–recapture models used to estimate the demographic parameters of the Lesser Antillean iguana population of Chancel Islet from 2012 to 2020

Model	Deviance	Num. Par	AICc	Delta AICc	AICc Weights
{phi(.) f(year) p(year+H)}	−9,852.31	37	3,062.90	0.00	0.82
{phi(year) f(year) p(year+H)}	−9,862.76	44	3,066.80	3.90	0.12
{phi(year+sex) f(year) p(year+H)}	−9,863.27	45	3,068.35	5.45	0.05
{phi(year+sex) f(year+sex) p(year+H)}	−9,861.70	46	3,071.97	9.07	0.01
{phi(year+sex) f(year+sex) p(H)}	−9,844.74	39	3,074.56	11.66	0.00
{phi(year+sex) f(year+sex) p(year+sex+H)}	−9,824.41	46	3,109.27	46.37	0.00
{phi(year+sex) f(.)p(year+H)}	−9,773.88	37	3,141.32	78.42	0.00
{phi(year+sex) f(year+sex) p(year)}	−9,767.87	44	3,161.69	98.79	0.00

Note

Phi is the survival probability; f, the recruitment rate; and P, the capture probability. Year means that a yearly variation was fitted on the parameter; sex, that an effect of sex was fitted; and H, that an effect of capture heterogeneity was fitted; “.” means the parameter was constant.

**FIGURE 2:**
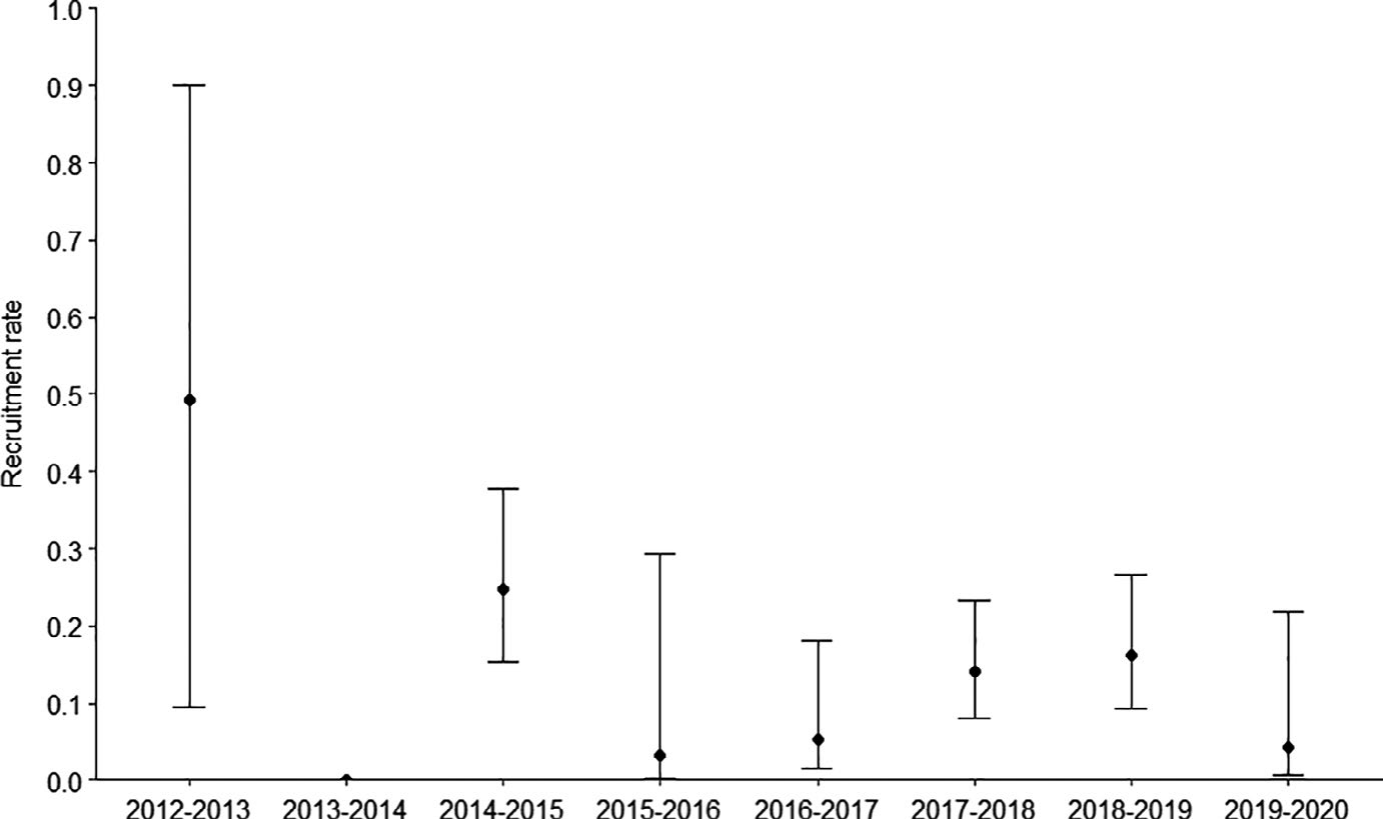
Yearly estimates of recruitment rate with 95% confidence intervals for the Lesser Antillean iguana population on Chancel Islet from 2012 to 2020

**FIGURE 3:**
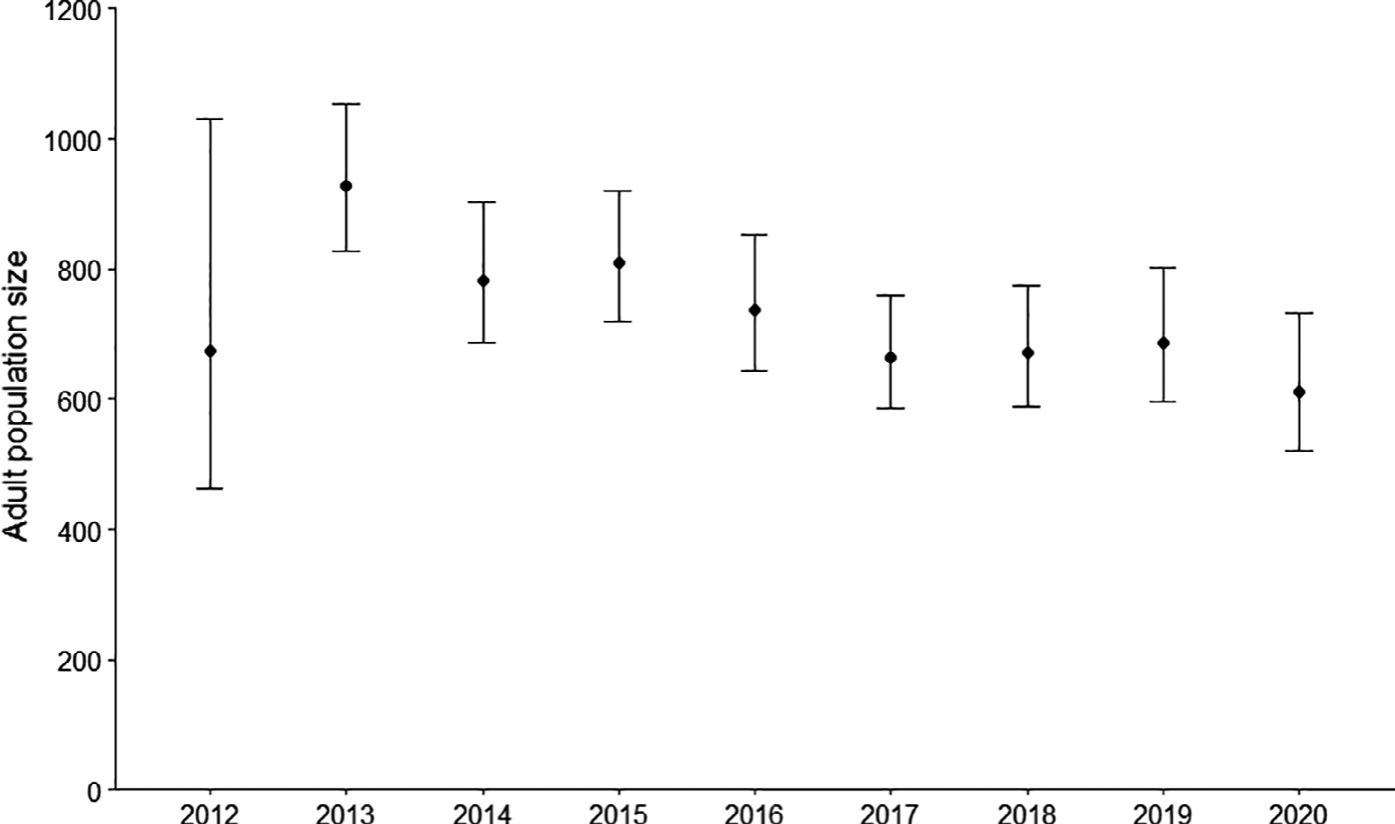
Yearly estimates of population size with 95% confidence intervals for the Lesser Antillean iguana population of Chancel Islet from 2012 to 2020

Models with temporal variability of survival probability had higher AICc than models with constant survival probability, suggesting that if among‐year variability of survival probability occurred, it was too low to be significant. The best model (Table 1) thus included constant survival probability, a recruitment rate that varied over years, and capture and recapture probabilities that varied over years with a heterogeneity effect. No effect of sex was retained. Only 0.08 [CI: 0.04–0.16] of the individuals were in the class of high recapture probability. For this class, daily capture probability ranged from 0.19 [CI: 0.12–0.30] to 0.36 [CI: 0.25–0.43], while daily capture probability was much lower for the rest of the population, ranging from 0.04 [CI: 0.03–0.07] to 0.10 [CI: 0.08–0.11] (Table S1). Survival probability was estimated at 0.850 [0.833–0.866]. Recruitment strongly varied over years, ranging from 0 to 0.492 [CI: 0.094–0.899] (Figure 2). Excluding the first year, which may be biased by the low number of marked individuals, we estimated that population abundance decreased from 928 [CI: 826–1053] in 2013 to 611 [CI: 519–730] in 2020 (Figure 3).

### 3.2 Population trajectory

**FIGURE 4:**
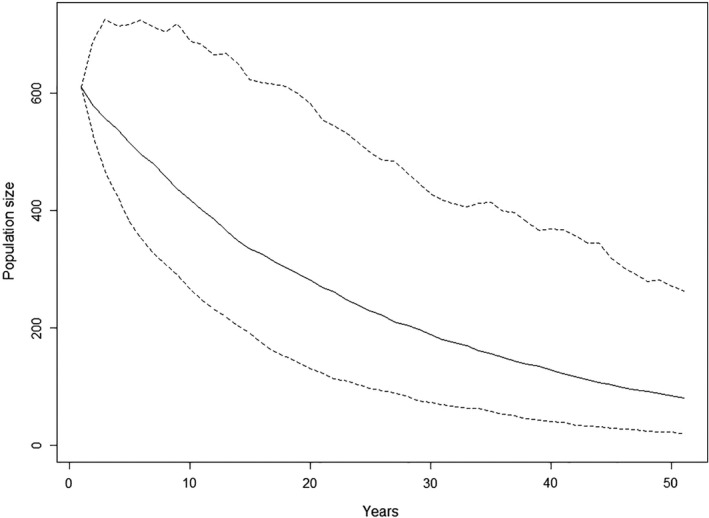
Predicted median population size with 95% confidence intervals of the Lesser Antillean iguana population of Chancel Islet over the next 50 years using the current recruitment rate estimates

Models based on the current demographic rates indicate that the population will rapidly decline (Figure 4). The median population size after 50 years was estimated to be about 80 individuals, at a mean annual population growth rate of 0.96. The probability that the population will decrease below a threshold of 50 individuals (quasi‐extinction) increases strongly after 40 years if the conditions remain the same, and reaches about 20% after 50 years.

**FIGURE 5:**
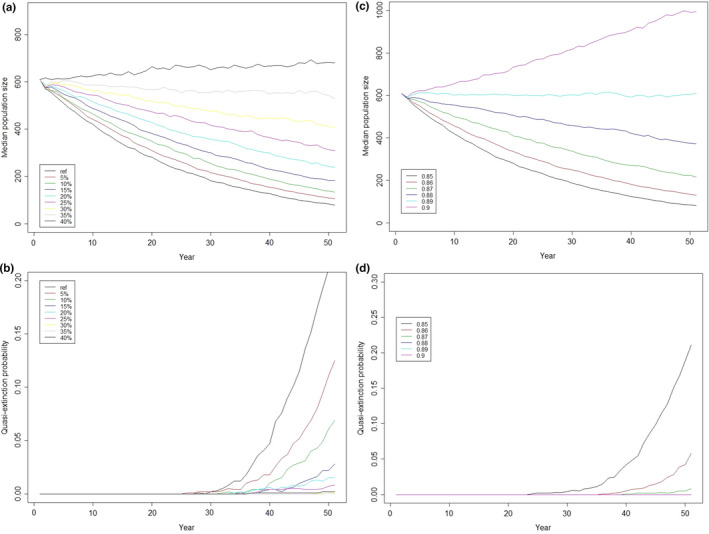
Predicted effects over the next 50 years if recruitment rates increased up to 40% by 5% increments on (a) the median population size and (b) the quasi‐extinction risk (population below 50 individuals). Predicted effects over the next 50 years if survival probability increased from 0.85 to 0.90 by 0.01 increments on (c) the median population size and (d) the quasi‐extinction risk

As expected, when the recruitment rate increased, the population trajectory improved (Figure 5a). Yet, the recruitment rate would have to improve by at least 40% (improving from 0.11 to 0.16 per individual present the year before) to allow the population size to remain stable or increase. To bring the probability of quasi‐extinction below 5% after 50 years, an increase of at least 10% in the recruitment rate is required (Figure 5b).

The population trajectory also improved when survival probability increased (Figure 5c). Survival probability would have to reach at least 0.89 to allow the population size to remain relatively stable. The probability of a population decrease below a threshold of 50 individuals was around 5% after 50 years when survival probability reached 0.86, and null when survival probability was above 0.88 (Figure 5d).

### 3.3 Effects of nesting site maintenance

Between 2012 and 2013 (before and after maintenance), female Lesser Antillean iguanas substantially increased their use of the three fenced nesting sites we monitored. For example, site #1 was abandoned in 2012, but at least 16 nests were counted on it in 2013. Interestingly, this site has a small slope and all 16 nests were placed on that slope. Sites #2 and #3 had 3 and 8 nests in 2012 versus 14 and 32 nests in 2013, respectively. Unlike in 2012, we did not observe any eggs scattered on the surface of the nesting sites in 2013. Hatching success also increased substantially, from 25% in 2012 (*N* = 36) to 84% in 2013 (*N* = 63). The observed predation rate decreased from 25% in 2012 (*N* = 36) to 14.5% in 2013 (*N* = 62). We provide a summary of the effects of site maintenance on nest and hatching success in Table 2.

**TABLE 2:** Data on nest and egg‐hatching success obtained before site maintenance (2012) and after (2013)

	2012	2013
Nest parameters		
Total number of nests identified	11	62
Proportion of nests with at least partial hatching	0.82	1
Number of nests we successfully excavated	3	5
Mean size of clutch + *SD* and range^a^	12.3 ± 1.7 [10–14]	12.6 ± 2.6 [9–17]
Egg parameters		
Total number of eggs retrieved	36	63
Number of eggs hatched	9	53
Number of eggs excavated	18	0
Number of eggs depredated	9	9
Unknown fate	0	1

^a^ Clutch size and range are given for the 3 nests successfully excavated in 2012 and the 5 successfully excavated in 2013.

## 4 DISCUSSION

Our study provides the first robust estimate of the abundance, adult survival, and recruitment rates of a Lesser Antillean iguana population. Although the density of this species on Chancel Islet (873.03 iguanas/km^2^ in 2020) is much higher than in other places (e.g., 35 iguanas/km^2^ in St Eustatius) (Debrot et al., 2013), it is still far below the highest estimated densities for this species, which can exceed 4,000 iguanas/km^2^ under favorable conditions (Knapp & Perez‐Heydrich, 2012). Despite the protected status of the islet and the lack of obvious threats to adult survival, the population declined rapidly at a rate of 4% per year, which would lead it to become quasi‐extinct within the next 50 years if drastic action is not taken to reverse the trend.

We observed low among‐year variability of adult survival probability over the past 10 years, and the PVA predicted that a small increase in adult survival (4%) would stabilize the population over the next five decades, whereas a 40% increase in recruitment would be necessary to reach the same effect. Due to its high elasticity, adult survival is strongly linked to the asymptotic population growth rate—this observation is a common feature among long‐lived species, including reptiles (Briggs‐Gonzalez et al., 2017; Gaillard & Yoccoz, 2003). Our results thus support the canalization hypothesis, as the parameter with the highest elasticity (i.e., adult survival) seems buffered against environmental variation (Gaillard & Yoccoz, 2003; Gibson & Wagner, 2000; Sæther & Bakke, 2000).

Yet because it only measures a proportional impact, elasticity alone does not determine how the different demographic parameters contribute to variation in the population growth rate and thus the population dynamics (Gaillard et al., 1998). The variance in a parameter may contribute as much or more to the population growth rate, due to the propensity of natural selection to stabilize a trait strongly related to fitness closer to its optimum (Pfister, 1998; Stearns & Kawecki, 1994). Our retrospective analysis suggests that the population trajectory of Chancel iguanas may have been more strongly influenced by the large variation over time we detected in recruitment than by constant adult survival, a pattern often found in other long‐lived species (Gaillard & Yoccoz, 2003; Sergio et al., 2020).

Adult survival was not only constant but also high (0.85), and consistent with results found in long‐lived lizards of similar size. For example, the survival rates were 0.86 and 0.90 in *Cyclura rileyi* and *C. cyclura*, respectively (Iverson et al., 2006, 2016), and 0.88 to 0.91 in *Amblyrhynchus cristatus* (Laurie & Brown, 1990). Elsewhere across its range, the main causes of adult mortality of the Lesser Antillean iguana are poaching, roaming dogs, road traffic, and fences, in which they can die of strangulation or dehydration (Debrot & Boman, 2013; Knapp et al., 2016). On Chancel Islet, however, there are no roads and no poaching, and although a few dogs are present, they can only range in a restricted area where almost no iguanas were captured. Over the last 23 years during which this population has been studied, no obvious threat to adult survival has been identified. Adult iguanas on the islet most likely die of natural causes. Thus, the seemingly small increase in adult survival of 4 points would in fact require an unrealistic reduction of almost 27% in adult mortality. This unrealistic goal supports our prediction that enhancing the recruitment rate would provide better leverage to halt or reverse the decline of this population.

The smallest increase in recruitment rate necessary to reverse the iguana population decline on Chancel Islet would require a substantial and sustained conservation effort (Figure 5b). Recruitment depends on multiple parameters, from reproductive success to the survival of the different immature cohorts (Gaillard et al., 1998; Sergio et al., 2020). In addition, long‐lived species usually mature late, at the age of 2 to 3 for the Lesser Antillean iguana (Knapp et al., 2014). This late maturation delays the observed effects of actions targeting either reproductive success or the survival of the youngest juveniles. Nest protection is a common strategy to improve the conservation status of reptiles, but the relationship between the number of hatchlings produced and the recruitment rate is not linear (Campbell et al., 2020). Unfavorable environmental conditions for juveniles may exert considerable pressure on their survival, negatively impacting the recruitment rate, even if several thousands of additional hatchlings enter the population yearly (Campbell et al., 2020). Unfortunately, our low capture rate of juveniles and lack of resources to monitor nests regularly prevented us from identifying which parameter affected recruitment the most or which cohort suffered the highest losses. Consequently, we argue that considering the high probability of quasi‐extinction within 50 years, conservation should target all stages that contribute to recruitment. Ideally, management actions should be backed by long‐term monitoring to better understand the effect of the different threats and assess the efficacy of each conservation action.

Despite the lack of a formal demographic study on the Chancel iguana population's nests and juveniles, results from pilot studies we carried out suggest that eggs, hatchlings, and juveniles of the Lesser Antillean iguanas all face threats linked to habitat degradation and predation (Curot‐Lodéon, 2015; Rodrigues, 2012). Reptile eggs and juveniles are both important food sources for many predators (Doody et al., 2009). In addition, eggs, considered by some the most vulnerable stage in reptiles, can suffer high losses from the lack of suitable nesting ground (Briggs‐Gonzalez et al., 2017; Eckert, 1987; Moss et al., 2020). These threats are common across reptiles in general (Bellard et al., 2016; Hailey et al., 2011; Knapp et al., 2014).

**FIGURE 6:**
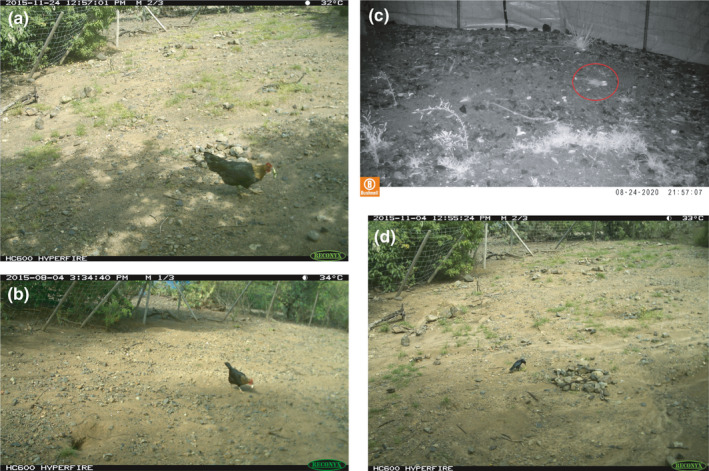
Hatchling and egg depredation by a domestic chicken (a) and (b), a black rat (c), and a Carib grackle (d) on Chancel's main nesting sites

We collected evidence using trail cameras and video systems on the three main nesting sites that diverse not only introduced predators— mainly black rats (*Rattus rattus*) and chickens (*Gallus gallus domesticus*)— but also endemic predators, such as Carib grackles (*Quiscalus lugubris*), preyed on eggs or juveniles (Curot‐Lodéon, 2015; Rodrigues, 2012) (Figure 6). We occasionally recorded the presence of small Indian mongooses, domestic cats (*Felis catus domesticus*), and green herons (*Butorides virescens*) on the nesting sites or elsewhere on the islet. Although we did not directly observe predation events by these animals, they are known predators of young reptiles or eggs (Case & Bolger, 1991). Ample evidence exists that eradication of introduced predators has substantial beneficial effects on the conservation and recovery of hundreds of endemic species, including critically endangered reptiles (Howald et al., 2007; Jones et al., 2016; Towns & Broome, 2003). Given the small size of Chancel Islet, diverse solutions to eradicate introduced predators are available and could be implemented easily and effectively. Live trapping has been used as a successful mongoose eradication technique on islets larger than Chancel (Lorvelec et al., 2004), and nearly six decades of documented rodent eradication campaigns have provided several efficient and cost‐effective strategies that can be applied on small islets (Towns & Broome, 2003; Veitch et al., 2019). Chickens are less commonly cited but known predators of immature reptiles. Relative to other predators—and despite their low numbers on the islet—chickens exerted high predation pressure on both eggs and juveniles, in addition to disturbing egg‐laying females (Video S1). Removing them became a priority, and the few on the islet were culled in 2015 (Curot‐Lodéon, 2015). Yet, predator eradication may not always be an option: for example, when predation pressure comes from endemic species and/or free‐roaming domestic animals of importance to the local population. In this case, eggs can be directly protected by using hardware cloth cages over individual nests, as has been done for some sea turtle populations (e.g., Lovemore et al., 2020), although such a protocol requires considerable effort, specifically during peak nesting season to identify a maximum number of nests. Moreover, hatchlings and juveniles are mobile organisms, and thus, in situ protection of these cohorts can only be done indirectly. Juveniles of diverse arboreal reptile species, unlike adults, use the understory to feed and to escape predators (Christian & Tracy, 1981; Henderson, 1974; Krysko et al., 2007). This habitat segregation may also lessen possible intraspecific competition for food and space with older cohorts (Henderson, 1974; Keren‐Rotem et al., 2006; Stamps, 1983). Thus, appropriate shrub cover is essential to juvenile survival and therefore to enhanced recruitment rates. Unfortunately, Chancel lacks understory over most of its surface area, likely due to high grazing pressure by the large flock of sheep (Figure S1). For these cohorts, predation mitigation will only be effective if habitat quality is improved in parallel.

Communal nesting is particularly widespread in lizards (Doody et al., 2009) and is associated with high costs, notably competition over space and resources and ovicide (Moss et al., 2020). As nest density increases, so does the probability of later‐laying females digging up earlier nests, thus scattering the eggs on the surface or in the burrow system (Moss et al., 2020; Rand, 1980). Ovicide may be exacerbated when suitable nesting space, which in many iguana species is open area, bare ground, and deep, loose, well‐drained soil, is limited (Krysko et al., 2007; Moss et al., 2020). For example, during a nesting survey of the Lesser Antillean iguana population of Dominica, Knapp et al. (2016) found that a minimum of 20% of eggs had been scattered outside of the nests and thus did not hatch. Soil erosion is severe on Chancel (Legouez et al., 2009). The positive effects on reproductive outputs of the site maintenance that took place during the pilot study we carried out in 2012 and 2013 suggested that soil erosion likely induced competition for nesting space. The strong decrease in the ovicide rate by competing nesting females and the high increase in hatching success observed following site maintenance may have led to the recruitment rate reaching almost 24% in 2014 (assuming 2 years to reach maturity). Our sample size and monitoring effort during the pilot study were limited, but similar for 2012 and 2013. While these results do not represent the true reproductive success, predation, and ovicide rates occurring on Chancel Islet, they are a proxy for the improved reproductive success that yearly maintenance of the nesting sites could achieve.

Based on this knowledge, we thus propose a three‐point plan to manage the threats posed by sheep: (a) remove sheep or only allow them access to a restricted area using enclosures or exclosures; (b) counter the effects of sheep grazing until the environment is restored by loosening the soil of nesting sites to provide females with suitable nesting space, and fencing important nesting sites to prevent roaming livestock from trampling nests; and (c) provide food and hiding places for hatchlings and juveniles using assisted vegetation restoration techniques near nesting sites, as prolonged grazing may render exclosures alone insufficient to allow vegetation to recover naturally (Baasch et al., 2012; Otsu et al., 2019). Although the three current main nesting sites on Chancel have been fenced off since 2006 and 2007, successfully preventing sheep from trampling the nests, no measures have been taken to prevent them from degrading the habitat. It would be valuable to conduct a complete search for nesting sites on Chancel, to fence off and maintain all sites as described above.

## 5 CONCLUSION

The key role of the most variable demographic rates in driving population dynamics is a growing focus of wildlife conservation and management—successful conservation plans need to treat populations as an integrated system of demographic rates limited by those most vulnerable to environmental conditions. To halt the decline of the studied population, our results indicate that directing conservation efforts toward the species' reproductive success and the survival of immature cohorts will be critical. As the threats we identified are widespread on islands (and elsewhere) and common to most reptiles and other taxa, the proposed solutions are thus likely applicable to most long‐lived reptile species and across taxa sharing common traits with our focal species.

## CONFLICT OF INTEREST

The authors declare no conflict of interest.

## AUTHOR CONTRIBUTIONS

**Chloé Warret Rodrigues:** Conceptualization (lead); Funding acquisition (supporting); Investigation (equal); Methodology (lead); Project administration (equal); Resources (equal); Supervision (equal); Visualization (lead); Writing‐original draft (lead); Writing‐review & editing (equal). **Baptiste Angin:** Funding acquisition (equal); Investigation (equal); Project administration (equal); Resources (lead); Supervision (equal); Writing‐original draft (supporting); Writing‐review & editing (equal). **Aurélien Besnard:** Conceptualization (lead); Formal analysis (lead); Funding acquisition (equal); Methodology (lead); Software (lead); Validation (equal); Writing‐original draft (supporting); Writing‐review & editing (equal).

## Supporting information

Appendix S1

Video S1

## Data Availability

Data are available from the Dryad Digital Repository: https://doi.org/10.5061/dryad.9p8cz8wgv.
