# Supplementary material for: Favoring recruitment as a conservation strategy to improve the resilience of long‐lived reptile populations: Insights from a population viability analysis
Source: Ecol Evol. 2021 Sep 15;11(19):13068–80. doi: 10.1002/ece3.8021 (PMC8495825; doi:10.1002/ece3.8021)
Supplement: Supplementary file 1 — Appendix S1 [file ECE3-11-13068-s001.docx]

**Appendices**

Table A1 – Yearly estimates of capture probability with 95% confident intervals for the two classes of individuals (with high or low capture probability).

| \| **Year** \| **Low cap. prob** \| **High capt. prob.** \| \| --- \| --- \| --- \| \| 2012 \| 0.04 [0.03-0.07] \| 0.19 [0.12-0.30] \| \| 2013 \| 0.09 [0.08-0.11] \| 0.34 [0.25-0.44] \| \| 2014 \| 0.08 [0.07-0.10] \| 0.32 [0.24-0.42] \| \| 2015 \| 0.10 [0.08-0.11] \| 0.36 [0.27-0.45] \| \| 2016 \| 0.08 [0.07-0.09] \| 0.31 [0.23-0.40] \| \| 2017 \| 0.10 [0.08-0.11] \| 0.36 [0.27-0.46] \| \| 2018 \| 0.09 [0.07-0.10] \| 0.33 [0.25-0.43] \| \| 2019 \| 0.08 [0.07-0.10] \| 0.31 [0.23-0.41] \| \| 2020 \| 0.07 [0.06-0.09] \| 0.28 [0.20-0.38] \| |
| --- | --- | --- | --- | --- | --- | --- | --- | --- | --- | --- | --- | --- | --- | --- | --- | --- | --- | --- | --- | --- | --- | --- | --- | --- | --- | --- | --- | --- | --- | --- |
|  |
|  |
|  |
|  |
|  |
|  |
|  |
|  |
|  |


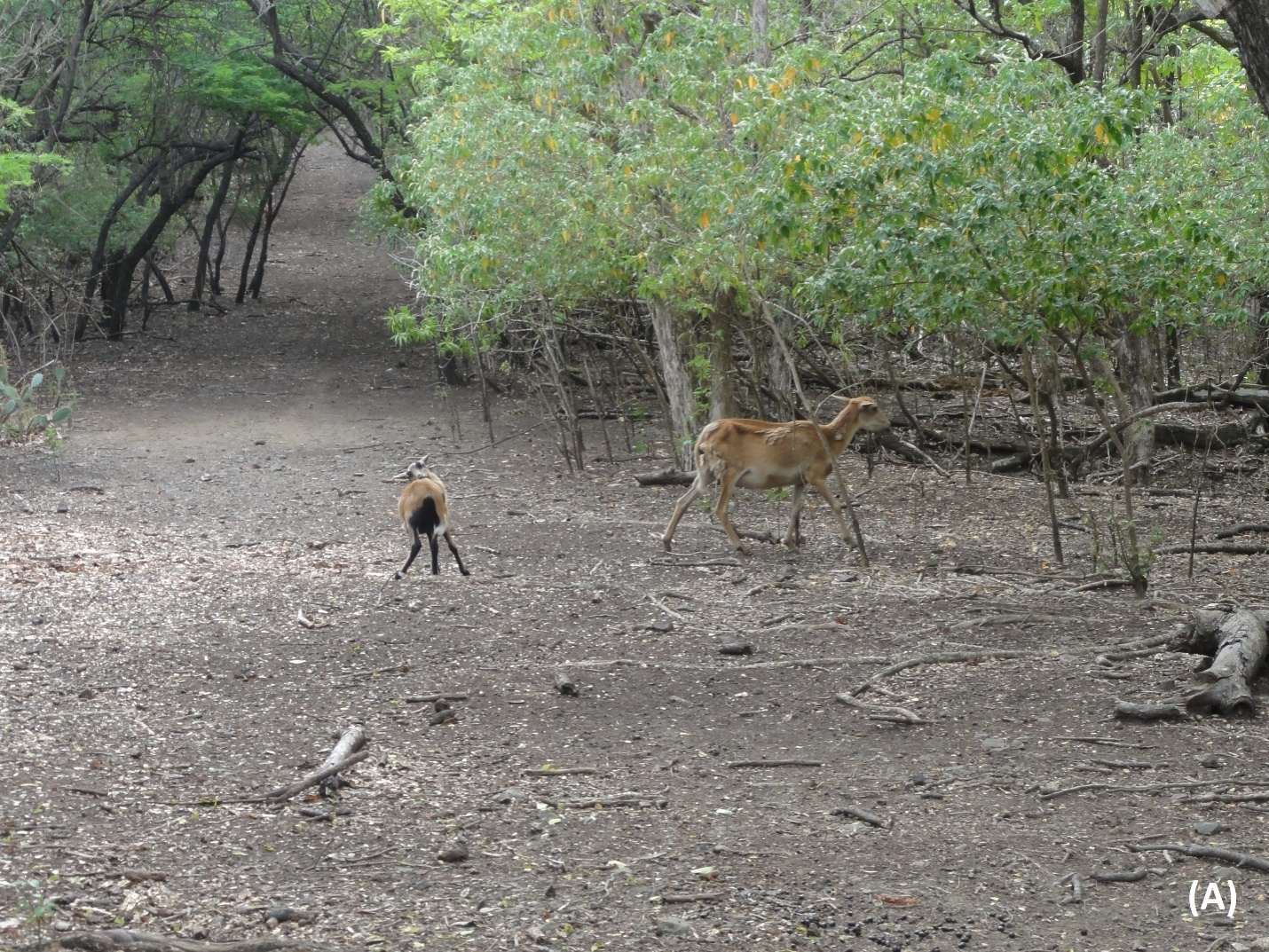

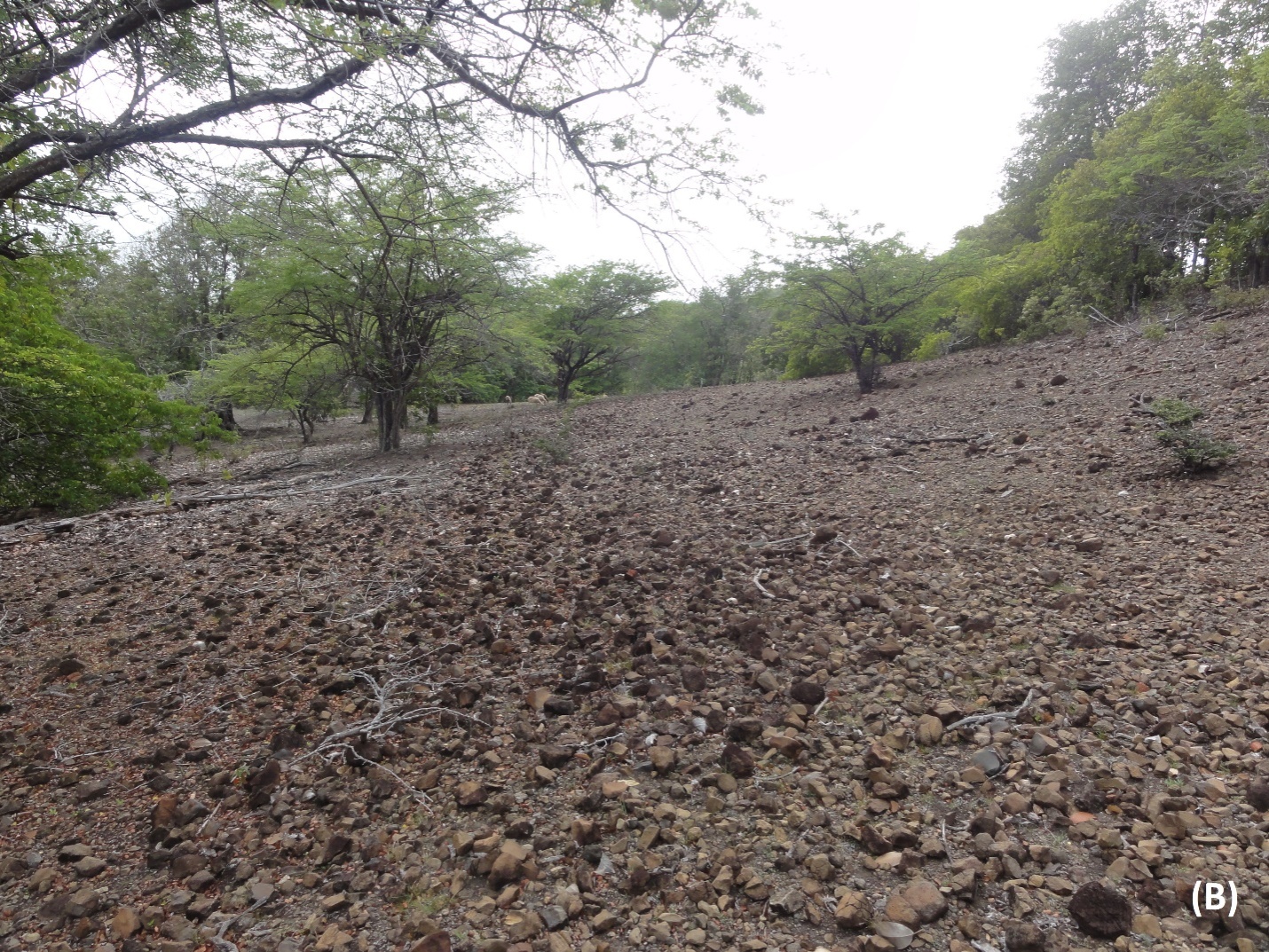


Figure A1 – Free-ranging sheep have overgrazed the islet, preventing forest regeneration (A) and leading to a lack of a vegetation understory (B).
